# Increased risk of herpes zoster with tofacitinib treatment in Korean patients with rheumatoid arthritis: a single-center prospective study

**DOI:** 10.1038/s41598-023-33718-7

**Published:** 2023-05-15

**Authors:** Yeo-Jin Song, Soo-Kyung Cho, Hyoungyoung Kim, Hye Won Kim, Eunwoo Nam, Ja-Young Jeon, Hyun-Jeong Yoo, Chan-Bum Choi, Tae-Hwan Kim, Jae-Bum Jun, Sang-Cheol Bae, Dae Hyun Yoo, Yoon-Kyoung Sung

**Affiliations:** 1 Department of Rheumatology, Hanyang University Hospital for Rheumatic Diseases, 222-1 Wangsimni-ro, Seongdong-gu, Seoul, 04763 Republic of Korea; 2 Hanyang University Institute for Rheumatology Research, Seoul, Republic of Korea; 3 Pfizer Pharmaceuticals Korea Ltd., Seoul, Republic of Korea

**Keywords:** Rheumatology, Infection, Epidemiology

## Abstract

We aimed to determine the risk of herpes zoster (HZ) in Korean rheumatoid arthritis (RA) patients on tofacitinib compared with tumor necrosis factor inhibitor (TNFi) treatment. From the prospective cohorts of RA patients who started tofacitinib or TNFi in an academic referral hospital in Korea, patients who started tofacitinib between March 2017 and May 2021 and those who started TNFi between July 2011 and May 2021 were included. Baseline characteristics of tofacitinib and TNFi users were balanced through inverse probability of treatment weighting (IPTW) using the propensity score including age, disease activity of RA and medication use. The incidence rate of HZ in each group and incidence rate ratio (IRR) were calculated. A total of 912 patients were included: 200 tofacitinib and 712 TNFi users. There were 20 cases of HZ among tofacitinib users and 36 among TNFi users during observation period of 331.4 person-years (PYs) and 1950.7 PYs, respectively. In IPTW analysis with a balanced sample, IRR of HZ was 8.33 (95% confidence interval 3.05–22.76). Tofacitinib use increased the risk of HZ compared with TNFi in Korean patients with RA, but the rate of serious HZ or permanent discontinuation of tofacitinib due to HZ event was low.

## Introduction

Tofacitinib is an oral Janus kinase inhibitor (JAKi) for the treatment of rheumatoid arthritis (RA)^1,2^, and was approved for RA treatment in Korea in 2015. Despite the convenient administration, there are concerns about the safety of tofacitinib, especially with regard to herpes zoster (HZ)^3,4^, which is caused by the reactivation of the varicella zoster virus (VZV) that remains dormant in sensory ganglia after a primary infection^5^.

Patients with RA have an increased risk of HZ, possibly due to immune-system dysregulation and the use of potent immunomodulatory drugs^6–8^. Old age and high disease activity of RA are known risk factors for HZ incidence among RA patients^9^. However, what clinicians are more interested in is whether disease-modifying anti-rheumatic drugs (DMARDs) affect the risk of HZ. The relationship between HZ incidence and methotrexate, the DMARD most commonly used for RA, has yet to be determined^10^. Some studies have reported an increased risk in patients using hydroxychloroquine but they emphasized the need for further study^11,12^.

An increased risk of HZ has been observed in RA patients treated with JAKi or certain biologic DMARDs (bDMARDs), such as monoclonal anti-tumor necrosis factor (TNF) antibodies and B cell targeted therapy, compared with those treated with conventional DMARDs^13^. Especially, the risk for HZ in tofacitinib-treated RA patients was higher than in bDMARD-treated RA patients^4,14^. Though the mechanism underlying the increase of HZ risk related to tofacitinib use is unclear, the tofacitinib-induced inhibition of the production, proliferation, and activation of interferon-γ are likely implicated in the poor VZV-specific cellular immune response^15^. Among tofacitinib users, both oral glucocorticoid use and Asian race were additional risk factors for HZ^16^.

Asian population was reported to be at higher risk of HZ, including Korean population^4^. Korea is one of the countries with typically high seroprevalence of VZV infection^17^. A previous study reported that 92.7% of young adults from the military personnel had serologic evidence of VZV infection, and were at risk for HZ^17^. A different study using claims data showed increasing disease burden of HZ in Korea, which was significantly higher than that in other countries^18^.

In Korea, two live zoster vaccines were available in the past: Zostavax^®^ and SKYzoster^®^. Live attenuated vaccines are not recommended for patients using immunosuppressants, so the zoster vaccination uptake in RA patients might have been low^19^. In September 2022, an inactivated recombinant zoster vaccine, Shingrix^®^, was approved in Korea, and was released in December 2022.

In Korea, there is a lack of real-world research data on the safety of tofacitinib use. This study aimed to determine whether tofacitinib use, compared with the use of TNF inhibitor (TNFi), increased the risk for HZ in Korean patients.

## Materials and methods

### Data collection and participants

Data were extracted from two prospective cohorts of RA patients at an academic referral hospital in Korea. Patients with RA satisfying the 1987 American College of Rheumatology (ACR) classification criteria or the 2010 ACR/European Alliance of Associations for Rheumatology (EULAR) classification criteria for RA were enrolled in the cohorts when initiating targeted therapy. All patients with RA who started targeted therapy in our institution were candidates for the cohorts, but those who refused to write an informed consent were excluded. In this study, patients with RA starting tofacitinib between March 2017 and May 2021 and those starting TNFi between July 2011 and May 2021 were included.

Demographic and clinical information of RA patients were collected at enrollment, and the disease activity was assessed every 6 months according to the Disease Activity Score 28-Erythrocyte Sedimentation Rate (DAS28-ESR) in combination with patient-reported outcomes, including the Health Assessment Questionnaire-Disability Index (HAQ-DI) and EuroQol-5 Dimensions Questionnaire (EQ-5D). The HAQ-DI was the mean of the eight category scores to assess arising, walking, dressing, hygiene, eating, reaching, gripping, and performing specific activities, rated on a scale ranging from 0 (without any difficulty) to 3 (unable to do)^20^. The EQ-5D was to assess mobility, self-care, usual activities, pain/discomfort, and anxiety/depression, with an index score between 0 (death) and 1 (perfect health)^20^.

Information on the occurrence of any adverse event (AE) during treatment was collected, and AEs were categorized and graded in accordance with the Common Terminology Criteria for Adverse Events (CTCAE) version 4.03. The medical records of patients were reviewed together to minimize the possibility of missing information on the study-related data points. Serious adverse events (SAEs), including AEs associated with death, hospitalization, disability, permanent damage, and birth defects, were also investigated.

### HZ case definition

An occurrence of HZ during treatment with tofacitinib or with TNFi was recorded as an AE (including SAE) and detailed information was extracted. The diagnosis of HZ was based on a clinician’s identification of typical skin lesions and clinical symptoms. A serious HZ case, defined as a case of HZ that was reported as a SAE or as of CTCAE Grade 3 or higher, were included. A HZ case reported as a SAE was a case requiring inpatient treatment or resulting in serious life-threatening disabilities and dysfunction. In addition, a HZ case of CTCAE Grade 3 or higher was a case requiring intravenous treatment or urgent intervention, or resulting in life-threatening consequences or death.

### Statistical analysis

To compare baseline characteristics between the tofacitinib and TNFi groups, we performed the Mann–Whitney *U* test for continuous variables and the Chi-Square test or Fisher’s exact test for categorical variables. In addition, the baseline features of tofacitinib and TNFi users were balanced through inverse probability of treatment weighting (IPTW) with the average treatment effect in the treated (ATT) based on propensity score (PS). We used multivariable logistic regression model, and age, sex, duration of RA, comorbidities, disease activity of RA, and medication use were included for calculation of the PS.

The observation period commenced from the initiation of tofacitinib or TNFi and continued until the onset of HZ, discontinuation of each agent, or May 2021. We calculated incidence rates (IR) of HZ as cases per 100 person-years (PYs). To compare the risk of HZ in tofacitinib users with that in TNFi users, incidence rate ratio (IRR) and 95% confidence interval (CI) were estimated using a Poisson regression model. The incidences of serious HZ were analyzed separately. In sensitivity analyses, the IRR of HZ development within 12 months of tofacitinib or TNFi use was calculated. Moreover, we also calculated the IRR of HZ in the study population after matching the inclusion periods (March 2017 to May 2021) of the two groups. The clinical characteristics of HZ cases among tofacitinib users were described in detail.

All analyses were performed using SAS^®^ 9.4 software (SAS Institute, Cary, NC, USA), and results were considered statistically significant when *p*-value was less than 0.05.

### Ethics statement

This study complies with the Declaration of Helsinki and was approved by the Institutional Review Board (IRB) of Hanyang university hospital (IRB no. HYUH 2016-08-037). For comparison, we used the existing prospective cohort data that have already been reviewed and approved by the IRB of our institution (IRB no. HYUH 2011-05-008). Written informed consent was obtained from all patients at the time of enrollment in each registry.

## Results

### Baseline characteristics

A total of 912 patients with RA were enrolled, and included 200 tofacitinib users and 712 TNFi users. The mean age of study population was 51.3 ± 13.2 years and 87.2% were female participants (Table 1). Tofacitinib users were older (53.4 ± 12.2 vs. 50.8 ± 13.5 years, *p* = 0.027) and had a longer duration of RA (10.8 ± 7.3 vs. 8.1 ± 7.9 years, *p* < 0.001) than TNFi users. Disease activity, which was assessed according to DAS28-ESR and physician’s global assessment, at enrollment was higher in tofacitinib users. However, the patient-reported outcome that was measured by HAQ-DI was worse in the TNFi group (1.7 ± 0.7 vs. 1.4 ± 0.7, *p* < 0.001). With regard to previous treatment before study enrollment, tacrolimus was used significantly more often in the tofacitinib group than in the TNFi group (43.0% vs. 23.7%, *p* < 0.001). The proportion of targeted therapy-naïve patients was larger in the TNFi group than in the tofacitinib group (85.8% vs. 48.0%, *p* < 0.001). Furthermore, participants in the TNFi group had a higher concomitant use of methotrexate (MTX; 90.7% vs. 84.5%, *p* = 0.011) and received a higher oral glucocorticoid dose (5.4 ± 2.9 vs. 5.0 ± 3.0 mg/day, *p* = 0.016) than participants in the tofacitinib group.

**Table 1:** Characteristics of study population.

Variables	Before IPTW			After IPTW		
	Tofacitinib	TNFi	*P*	Tofacitinib	TNFi	*P*
	(n = 200)	(n = 712)		(n = 200)	(n = 200)	
Sex, female	181 (90.5)	614 (86.2)	0.111	181 (90.5)	186 (93.2)	0.326
Age, years	53.4 ± 12.2	50.8 ± 13.5	0.027	53.4 ± 12.2	53.6 ± 7.2	0.767
Disease duration of RA, years	10.8 ± 7.3	8.1 ± 7.9	< 0.001	10.8 ± 7.3	11.6 ± 4.7	0.186
BMI, kg/m^2^ (n = 200, 445, 200, 200)	22.3 ± 3.6	22.4 ± 3.7	0.854	22.3 ± 3.6	22.6 ± 2.1	0.306
Comorbidities						
Diabetes mellitus	22 (11.0)	54 (7.6)	0.123	22 (11.0)	22 (10.8)	0.946
Chronic pulmonary disease	2 (1.0)	40 (5.6)	0.006	2 (1.0)	18 (9.0)	< 0.001
Mild liver disease	9 (4.5)	23 (3.2)	0.389	9 (4.5)	4 (1.8)	0.121
Solid tumor	9 (4.5)	12 (1.7)	0.030	9 (4.5)	2 (0.9)	0.026
Cerebrovascular disease	1 (0.5)	4 (0.6)	0.999	1 (0.5)	3 (1.6)	0.284
Renal disease	5 (2.5)	11 (1.5)	0.364	5 (2.5)	11 (5.4)	0.132
Congestive heart failure	2 (1.0)	3 (0.4)	0.303	2 (1.0)	0 (0.2)	0.265
Peripheral vascular disease	1 (0.5)	2 (0.3)	0.525	1 (0.5)	0 (0.3)	0.679
CCI score^a^	0.4 ± 0.8	0.3 ± 0.7	0.147	0.4 ± 0.8	0.4 ± 0.5	0.594
RA disease activity						
DAS28-ESR	6.5 ± 0.8	6.3 ± 0.9	< 0.001	6.5 ± 0.8	6.5 ± 0.5	0.955
Physicians’ Global Assessment of Disease activity, VAS	76.0 ± 13.9	67.0 ± 17.1	< 0.001	76.0 ± 13.9	70.5 ± 9.2	< 0.001
Patient-reported outcomes						
Patient’s Global Assessment of Disease Activity, VAS	79.8 ± 13.1	73.8 ± 18.1	< 0.001	79.8 ± 13.1	80.4 ± 8.2	0.532
HAQ-DI (n = 200, 710, 200, 200)	1.4 ± 0.7	1.7 ± 0.7	< 0.001	1.4 ± 0.7	1.5 ± 0.4	0.678
EQ-5D (n = 200, 710, 200, 200)	0.6 ± 0.2	0.6 ± 0.2	0.101	0.6 ± 0.2	0.6 ± 0.1	0.890
Laboratory test						
Either RF or ACPA positivity	183 (91.5)	658 (92.4)	0.669	183 (91.5)	175 (87.7)	0.212
ESR, mm/h	56.1 ± 28.8	58.4 ± 28.9	0.326	56.1 ± 28.8	57.0 ± 14.6	0.696
CRP, mg/dL	2.4 ± 2.9	2.8 ± 3.0	0.011	2.4 ± 2.9	2.4 ± 1.4	0.936
Medications, ever use						
Conventional synthetic DMARDs						
Methotrexate	199 (99.5)	704 (98.9)	0.692	199 (99.5)	198 (99.0)	0.623
Hydroxycholoroquine	113 (56.5)	504 (70.8)	< 0.001	113 (56.5)	127 (63.7)	0.140
Sulfasalazine	124 (62.0)	449 (63.1)	0.784	124 (62.0)	115 (57.5)	0.356
Leflunomide	133 (66.5)	430 (60.3)	0.116	133 (66.5)	132 (66.1)	0.933
Tacrolimus	86 (43.0)	169 (23.7)	< 0.001	86 (43.0)	36 (18.2)	< 0.001
Number of conventional synthetic DMARDs, ever use	3.5 ± 1.0	3.5 ± 1.2	0.896	3.5 ± 1.0	3.6 ± 0.7	0.149
Number of previously used targeted therapies			< 0.001			0.134
0	96 (48.0)	611 (85.8)		96 (48.0)	94 (47.2)	
1 or 2	90 (45.0)	100 (14.0)		90 (45.0)	100 (50.0)	
≥ 3	14 (7.0)	1 (0.1)		14 (7.0)	6 (2.9)	
Targeted therapy, current use						
TNFi						
Adalimumab		294 (41.3)	NC		57 (28.7)	NC
Etanercept		261 (36.6)	NC		75 (37.8)	NC
Golimumab		71 (10.0)	NC		43 (21.4)	NC
Infliximab		86 (12.1)	NC		24 (12.2)	NC
JAK inhibitor						
Tofacitinib	200 (100.0)		NC	200 (100.0)		NC
Concomitant medications						
Methotrexate	169 (84.5)	647 (90.7)	0.011	169 (84.5)	170 (85.3)	0.827
Methotrexate dose, mg/week	11.5 ± 2.8	13.4 ± 2.7	< 0.001	11.5 ± 2.8	13.1 ± 1.5	< 0.001
NSAIDs	165 (82.5)	589 (82.7)	0.941	165 (82.5)	164 (81.8)	0.861
Oral glucocorticoid	153 (76.5)	579 (81.2)	0.130	153 (76.5)	150 (75.0)	0.720
Glucocorticoid dose^b^**,** mg/day	5.0 ± 3.0	5.4 ± 2.9	0.016	5.0 ± 3.0	5.2 ± 1.9	0.425

Data were presented in the form of number with percentage (%) or mean ± standard deviation.

*IPTW* Inverse probability of treatment weighting, *TNFi* Tumor necrosis factor inhibitor, *RA* Rheumatoid arthritis, *BMI* Body mass index, *CCI* Charlson Comorbidity Index, *DAS* Disease activity score, *ESR* Erythrocyte sedimentation rate, *CRP* C-reactive protein, *VAS* Visual Analogue Scale, *HAQ-DI* Health assessment questionnaire disability index, *RF* Rheumatoid factor, *ACPA* Anti-citrullinated protein antibody, *DMARD* Disease-modifying anti-rheumatic drug, *NSAID* Non-steroidal anti-inflammatory drug, *NC* Not calculated.

^a^The number of comorbidities included in the calculation of CCI, with the exception of rheumatoid arthritis, is presented; connective tissue diseases were excluded from the calculation of the CCI.

^b^Prednisolone-equivalent dose of oral glucocorticoid is presented.

Differences in the baseline variables, including age, duration of RA, initial disease activity, number of previously used targeted therapy, concomitant MTX use, and oral glucocorticoid dose, were balanced after IPTW between 200 tofacitinib users and 200 TNFi users.

### Incidence rate of HZ in RA patients treated with tofacitinib versus TNFi

There were 20 and 36 HZ cases among tofacitinib and TNFi users during observation periods of 331.4 and 1950.7 PY, respectively (Table 2). The IRR before IPTW was 3.27 (95% CI 1.89–5.65, *p* < 0.001). In the balanced sample after IPTW, the IRs of HZ were 6.03 and 0.77 cases per 100 PY in the tofacitinib and TNFi groups, respectively. The IRR for HZ development was 8.33 (95% CI 3.05–22.76, *p* < 0.001). For serious HZ cases, the IRR was 3.52, but without statistical significance (95% CI 0.14–93.51, *p* = 0.452).

**Table 2:** Incidence of HZ in RA patients treated with tofacitinib versus those treated with TNFi.

	Tofacitinib		TNFi		Incidence rate ratio^b^ (95% CI)	*P*
	Case	Incidence rate^a^	Case	Incidence rate^a^		
Before IPTW						
HZ	20	6.03	36	1.85	3.27 (1.89–5.65)	< 0.001
Serious HZ^c^	1	0.30	6	0.31	0.99 (0.12–8.20)	0.987
After IPTW						
HZ	20	6.03	5	0.77	8.33 (3.05–22.76)	< 0.001
Serious HZ^c^	1	0.30	1	0.15	3.52 (0.14–93.51)	0.452

*HZ* herpes zoster, *RA* rheumatoid arthritis, *TNFi* tumor necrosis factor inhibitor, *CI* confidence interval, *IPTW* inverse probability of treatment weighting, *SAE* serious adverse event, *CTCAE* Common Terminology Criteria for Adverse Events.

^a^Incidence rate was calculated as cases per 100 person-years.

^b^Incidence rate ratio and 95% CI were estimated using a Poisson regression model.

^c^Serious HZ was defined as a case that was reported as a SAE or with Grade 3 or more severity according to the CTCAE version 4.03.

In the sensitivity analysis, the IRR of HZ development within 12 months increased significantly after IPTW to 10.83 (95% CI 1.58–74.51, *p* = 0.015) (Table 3). The IRR of HZ in the study population after matching the inclusion periods in two groups are presented in Supplementary Table 1.

**Table 3:** Incidence of HZ within 12 months of tofacitinib or TNFi use.

	Tofacitinib		TNFi		Incidence rate ratio^b^ (95% CI)	*P*
	Case	Incidence rate^a^	Case	Incidence rate^a^		
Before IPTW	12	7.39	9	1.61	4.59 (1.93–10.88)	< 0.001
After IPTW	12	7.39	1	0.60	10.83 (1.58–74.51)	0.015

*HZ* Herpes zoster, *TNFi* Tumor necrosis factor inhibitor, *CI* Confidence interval, *IPTW* Inverse probability of treatment weighting.

^a^Incidence rate was calculated as cases per 100 person-years.

^b^Incidence rate ratio and 95% CI were estimated using a Poisson regression model.

### Characteristics of HZ cases among tofacitinib users

A total of 20 patients developed HZ during tofacitinib treatment, with ages ranging from 35 to 72 years. Of these, 16 (80%) were in their 50 s or 60 s, and 12 (60%) developed HZ within 1 year of starting tofacitinib. HZ led to permanent discontinuation of tofacitinib in only one case. Sixteen patients (80%) used MTX concomitantly, and 10 (50%) were receiving oral glucocorticoids. Moreover, 60% of these patients were targeted therapy-naïve, and only one patient had been vaccinated against HZ prior to the initiation of tofacitinib treatment (Table 4).

**Table 4:** Characteristics of HZ cases among tofacitinib users.

	Age	Sex	Duration of tofacitinib use until HZ development (month)	Discontinuation of tofacitinib	Serious HZ	MTX use	MTX dose (mg/week)	Oral GC use	Oral GC dose (prednisolone-equivalent, mg/day)	History of targeted therapy	HZ vaccination
Case 1	62	F	1.8	N	N	Y	10	Y	1.3	N	N
Case 2	61	F	2.7	N	N	Y	7.5	N	NA	N	N
Case 3	35	F	2.7	N	N	Y	12.5	Y	5	Y	N
Case 4	59	F	3	N	N	Y	12.5	Y	7.5	Y	N
Case 5	70	F	4.5	N	N	Y	12.5	Y	2.5	Y	N
Case 6	68	F	4.7	N	N	Y	10	N	NA	N	N
Case 7	72	F	5.1	N	N	Y	15	Y	2.5	N	N
Case 8	57	F	6.7	N	N	N	NA	N	NA	N	N
Case 9	54	F	6.8	N	N	N	NA	N	NA	N	N
Case 10	68	F	9.9	N	N	Y	12.5	Y	5	N	N
Case 11	58	M	10.5	N	N	N	NA	N	NA	Y	N
Case 12	58	F	11.2	N	N	Y	10	Y	5	Y	Y
Case 13	54	F	12.2	N	N	Y	12.5	Y	3.8	N	N
Case 14	66	F	12.6	N	N	Y	5	N	NA	N	N
Case 15	51	F	19.6	N	Y	Y	10	N	NA	N	N
Case 16	45	F	21.5	Y	N	Y	7.5	Y	5	Y	N
Case 17	59	F	28.9	N	N	N	NA	N	NA	Y	N
Case 18	60	F	32.1	N	N	Y	7.5	N	NA	Y	N
Case 19	59	F	36.5	N	N	Y	10	N	NA	N	N
Case 20	65	M	43.6	N	N	Y	15	Y	2.5	N	N

*HZ* Herpes zoster, *MTX* Methotrexate, *GC* Glucocorticoid, *NA* Not applicable.

## Discussion

In this single-center cohort study, there were several differences in the characteristics of tofacitinib users and TNFi users including age, duration of RA, disease activity, and medication use. After balancing the different features through IPTW, tofacitinib users had a higher risk of HZ than TNFi users. However, with regard to serious HZ cases, there was no significant intergroup difference and the number of serious HZ cases was small among tofacitinib users.

Studies have investigated the risk of HZ among tofacitinib users. According to a long-term study of tofacitinib for up to 9.5 years, the IR of HZ was 3.13 per 100 PYs in tofacitinib users^21^. Most of the HZ cases (96%) were non-serious, and 30 of the total 526 HZ cases had recurrent HZ. In a recent cohort study that investigated the comparative safety of tofacitinib and bDMARDs using data from the US CorEvitas (formerly Corrona) RA Registry, the risk of HZ was significantly higher with tofacitinib versus bDMARD use (adjusted hazard ratio [HR] 2.32, 95% CI 1.43–3.75)^22^. There was no serious HZ case among the tofacitinib users whereas five serious HZ cases were reported among 8,358 bDMARD users. The results from the ORAL Surveillance, post-marketing study showed that, compared with TNFi users, patients who received tofacitinib showed an increased risk of HZ, in both the 5 and 10 mg twice-daily dosage groups (HR 3.28, 95% CI 2.44–4.41 and HR 3.39, 95% CI 2.52–4.55)^23^. One HZ case led to permanent discontinuation of tofacitinib in this study. The IRR of HZ in tofacitinib users, when compared with that in TNFi users, was considerably higher than in previous studies, and increased markedly within 12 months. It should be considered that the rate of vaccination against HZ among tofacitinib users was low, but a comparison could not be undertaken due to the lack of information on the HZ vaccination status of TNFi users.

The Asian race is a risk factor of HZ development in RA patients^4^. In a post hoc analysis that included pooled data from RA patients in the Asia–Pacific region who were treated with tofacitinib, the IR of HZ was 5.9 per 100 PYs (95% CI 5.2–6.7)^24^, which was similar to the result of our study (IR 6.03 per 100 PYs). In the same study, the global IR of HZ was 3.8 per 100 PYs (95% CI 3.5–4.1), which was significantly lower than the IR of HZ among Asian patients. The reason for the increased HZ risk in Asian patients is unclear, though a recent meta-analysis of genome-wide association study suggested that a single-nucleotide polymorphism near *IL17RB* was associated with a predisposition to a faster onset of HZ, especially in East Asian subjects^25^.

In our study, 60% of HZ cases in the tofacitinib group developed HZ within 1 year of initiating tofacitinib treatment. Furthermore, the IRR of HZ development within 12 months of treatment initiation significantly increased in tofacitinib users compared with TNFi users. Therefore, close monitoring to detect HZ onset is necessary, especially during the first year of tofacitinib user.

Oral glucocorticoid use is a known risk factor for HZ development, whereas the independent effect of MTX use on HZ incidence is controversial^6,16,26^. In this study, only half of the HZ cases were concomitantly receiving low-dose oral glucocorticoids, and the proportion was even lower than in the total study population. Though the proportion of patients who were using concomitant MTX was as high as 80% among HZ cases in the tofacitinib group, this was similar to the proportion in whole study population. Moreover, the results of our previous study identified high disease activity as a risk factor for HZ^27^, suggesting that not only medication use but disease activity of RA would have influenced HZ onset.

Our study had some strengths. First, we used data from a well-established cohort, including information about RA disease activity and medication use. Therefore, we were able to balance characteristics between tofacitinib users and TNFi users by IPTW using the PS. Since patients were enrolled and followed from the time of initiating tofacitinib / TNFi, immortal time bias is unlikely to have occurred in this study design. Furthermore, detailed information about the characteristics of HZ cases in the tofacitinib group was available. Second, our study analyzed Korean RA patients, who are well-known to have a high incidence of HZ. The findings from this study supplemented the evidence for the increased risk of HZ in tofacitinib users, especially Asian patients on whom there is a relative paucity of real-world data.

Nonetheless, this study had several limitations. First, a history of HZ was not considered in our study due to the absence of information on this aspect in the cohort. We could only determine the incidence of HZ during tofacitinib or TNFi use. Second, vaccination against HZ could not be compared between the two groups or be included in the PS because this characteristic was only investigated in tofacitinib users. However, the rate of HZ vaccination was very low, as determined in our previous study^27^; therefore, the impact of vaccination on the results might not be strong. Third, the observation period of tofacitinib users was shorter than that of TNFi users because tofacitinib was only recently approved for RA treatment. However, the result of sensitivity analysis for HZ development within 12 months supported the main outcome. Further studies would be informative to analyze data from a larger cohort and for a longer observation period. Fourth, the number of serious HZ events was too small to be compared between the two groups, especially in the tofacitinib group. Though the IRR of serious HZ was increased, the wide 95% CI and the rarity of serious HZ event should be considered.

## Conclusion

In conclusion, this study showed that tofacitinib users were at an increased risk for HZ than TNFi users even after adjusting for different baseline characteristics among the patient groups. The incidence of serious HZ was also increased in tofacitinib users but not statistically significant, and the probability of permanent discontinuation of tofacitinib due to HZ event was low. Appropriate education and reassurance about the HZ risk for RA patients who are initiating tofacitinib treatment would help improve early detection and treatment of HZ as well as appropriate discontinuation of tofacitinib.

## Supplementary Information

Supplementary Table S1.

## Data Availability

Data are available upon request as the research data contain sensitive and potentially patient-identifying information. Requests for data and queries pertaining to the study should be directed to the corresponding author.
