# Supplementary material for: Increased risk of herpes zoster with tofacitinib treatment in Korean patients with rheumatoid arthritis: a single-center prospective study
Source: Sci Rep. 2023 May 15;13:7877. doi: 10.1038/s41598-023-33718-7 (PMC10185531; doi:10.1038/s41598-023-33718-7)
Supplement: Supplementary file 1 — Supplementary Table S1. [file 41598_2023_33718_MOESM1_ESM.pdf]

Increased Risk of Herpes Zoster with Tofacitinib Treatment in Korean Patients with Rheumatoid Arthritis: A Single-Center Prospective Study

Yeo-Jin Song[1,2], Soo-Kyung Cho[1,2], Hyoungyoung Kim[1,2], Hye Won Kim[2], Eunwoo Nam[2], Ja-Young Jeon[3], Hyun-Jeong Yoo[3], Chan-Bum Choi[1,2], Tae-Hwan Kim[1,2], Jae-Bum Jun[1,2], Sang-Cheol Bae[1,2], Dae Hyun Yoo[1,2], Yoon-Kyoung Sung[1,2]

[1] Department of Rheumatology, Hanyang University Hospital for Rheumatic Diseases, Seoul, Republic of Korea

[2] Hanyang University Institute for Rheumatology Research, Seoul, Republic of Korea

[3] Pfizer Pharmaceuticals Korea Ltd., Seoul, Republic of Korea

Supplementary Table 1. Incidence of HZ in RA patients treated with tofacitinib versus those treated with TNFi between March 2017 and May 2021

	**Tofacitinib**		**TNFi**		**Incidence rate ratio[b] (95% CI)**	***P***
	**Case**	**Incidence rate[a]**	**Case**	**Incidence rate[a]**		
Before IPTW						
**Total observation period**	20	6.03	9	2.57	2.35 (1.07–5.16)	0.033
**Within 12 months**	12	7.39	4	2.37	3.12 (1.01–9.66)	0.049
After IPTW						
**Total observation period**	20	6.03	2	0.94	6.45 (1.40–29.75)	0.017
**Within 12 months**	12	7.39	1	0.81	9.17 (1.08–77.73)	0.042

HZ herpes zoster, *RA* rheumatoid arthritis, *TNFi* tumor necrosis factor inhibitor, *CI* confidence interval, *IPTW* inverse probability of treatment weighting.

[a] Incidence rate was calculated as cases per 100 person-years.

[b] Incidence rate ratio and 95% CI were estimated using a Poisson regression model.
